# Inhaler Non-Adherence, Associated Factors and Asthma Control among Asthma Patients in a Tertiary Level Hospital in Tanzania

**DOI:** 10.24248/eahrj.v6i1.682

**Published:** 2022-07

**Authors:** Grace A. Shayo, Amina Omary, Ferdinand Mugusi

**Affiliations:** a Pulmonology section of the Department of internal medicine, Muhimbili University of Health and Allied Sciences, Dar es Salaam Tanzania.

## Abstract

**Background:**

Inhaled medications including corticosteroids are the most effective long-term controller medicines for asthma-related chronic airway inflammation. Despite this fact, 30% to 70% of the uncontrolled asthma patients report non-adherence to their inhalers. This study investigated factors affecting inhaler non-adherence among outpatient asthma patients in Muhimbili National Hospital, Dar es Salaam Tanzania and related the level of inhaler adherence to the extent of asthma control.

**Methods:**

A cross-sectional hospital-based study was conducted among patients with bronchial asthma in the pulmonology clinic of Muhimbili National Hospital in Dar-es-salaam, Tanzania. Patients' demographic, clinical and socio-economic factors were collected using a structured questionnaire. Medication adherence was self-reported using a 10-item Test of Adherence to Inhalers (TAI) questionnaire. Adherence was gauged as good when the score was 50, intermediate (score 46-49) or poor (score ≤ 45). Asthma control was assessed using a 5-question Asthma Control Test (ACT). A score of ≥20 meant well controlled asthma while a score of ≤19 meant poorly controlled asthma. Patients' inhaler use technique was assessed using a 10-step checklist. Patient's technique was regarded correct when all the steps were performed correctly. Categorical data were summarised as proportions. Binary logistic regression was performed to identify factors associated with inhaler non-adherence. Significance level was set at p-value less than .05.

**Results:**

A total of 385 asthma patients were enrolled in the study. Females were 206 (53.5%), 232(60.3%) were non-adherent to medications and 283(73.5%) had poorly controlled asthma. Lack of health insurance, fear of medication side effects, being too busy, having alternative medication for asthma and incorrect inhaler technique were significantly associated with non-adherence to inhalers, all p-values <.05.

**Conclusion:**

The magnitude of inhaler non-adherence and poorly controlled asthma were very high. Promoting adherence through patients' education on asthma and its management, emphasis on patients' insurance coverage and setting aside time to care for ones' self are fundamental in optimising asthma care and treatment.

## BACKGROUND

The Global initiative for asthma (GINA) defines asthma as a heterogeneous disease characterised by chronic airway inflammation. Asthma disease definition relies upon a history of respiratory symptoms such as wheeze, shortness of breath, chest tightness and cough that vary over time in intensity, together with variable expiratory airflow limitation.^1^ The long-term goals of asthma management are; to achieve good control of the symptoms, maintaining normal activity levels, minimise future risk of exacerbations, fixed airflow and limitation of side-effects from asthma medications.^2^ To achieve these goals, inhaled asthma medications are preferred over oral or parental medications. Pharmacologically, two groups of inhaled medications are used. The first group comprises of reliever medications that comprise of short acting beta agonists and short acting muscarinic antagonists. The second group comprises of long-term controller or preventer medicines that comprise of inhaled corticosteroids, long acting beta agonists, long acting muscarinic antagonists and their add-on therapies that consist of oral corticosteroids, leukotriene receptor antagonists and methylxanthines.^2^ Clinically, inhaled medications reduce the severity of asthma symptoms; improve peak flow measurements and other measures of lung function. Corticosteroids specifically prevent exacerbations and possibly prevent long-term lung remodeling.^3^ Despite the availability of these highly effective pharmacotherapy, non-controlled asthma is present in 70 to 95% of patients in Western Europe and the Asian-Pacific region.^4^ Literature on asthma control is scarce in African countries including Tanzania despite the fact that asthma cases are on the rise in Africa. It is estimated that asthma prevalence has increased in Africa from 94.8 million in the year 2000 to 119.3 million in the year 2010.^5^ In Tanzania, a 2007 study among urban and rural pupils reported a significantly higher prevalence of asthma in an urban region compared to a rural district. More pupils (23.1%) in the urban district reported a wheeze in the past 12 months compared to 12.1% in the rural district. In the same study, self-reported asthma was found in 17.6% and 6.4% of pupils in the urban and rural districts respectively, while exercise-induced asthma was significantly higher in urban (26.3%) in rural district (2.4%).^6^

Adherence to a medication regimen is generally defined as the extent to which the amount of medication patients take corresponds with agreed recommendations from a health care provider.^7,8^ Patients with persistent asthma are recommended to use preventer medicine daily throughout the year so as to keep their asthma under control.^9,10^ If a patient uses his inhaler once daily instead of the recommended two doses per day, adherence is 50%, and lower than 50% if medications are taken only when symptoms are present. Typical adherence rates for prescribed medications are about 50%, being 30% to 70% among patients with asthma.^10^ Medication non adherence is among the factors reported to affect asthma control in numerous previous studies.^11–13^

Inhaler non-adherence has been linked to various factors, both modifiable and non-modifiable. Non-modifiable factors are such as long duration of medication use, the use of multiple medications mostly delivered as inhalation, and the periods of symptoms remission.^2,7,9^ Modifiable factors include difficulties in use of inhaler devices, misunderstanding or lack of instruction,^14^ complex medication regimens, dissatisfaction with healthcare professionals, medication side effects, medication costs, inappropriate expectations, poor supervision, lack of training or follow-up, and forgetfulness.^15–17^ Patient's dislike of medication, fears about side effects, underestimation of disease severity and cultural or religious issues have been mentioned as contributing to non-adherence.^15–17^ Socio-demographic factors linked to non-adherence to asthma medications include; lower level of education^18^ and age below 50 years.^19^ However, non-adherence has also been observed in advanced age^20^ probably owing to memory difficulties which predispose patients to forget to take their medication. Furthermore, the elderly are often receiving treatment for other chronic health conditions thus having medication burden. Health system inadequacies may be pivotal to both poor medication adherence and asthma control. In a secondary data analysis study to explore the availability of services and level of health facilities readiness to provide management of chronic respiratory diseases and its associated factors using the 2014 to 2015 Tanzania Service Provision Assessment Survey data, it was reported that only about 10% of the facilities had high readiness to provide management of chronic respiratory diseases.^21^ A similar study to explore readiness of healthcare facilities in Sub-Sahara Africa to manage chronic respiratory disease found that only one health facility in Tanzania and 5 in Sudan, attained a Chronic Lung Disease (CLD) readiness score of ♕ 50% for CLD care.^22^

This study was conducted to investigate asthma control, inhaler non-adherence and factors affecting adherence in a Tanzanian context.

## METHODS

### Study Design, Site and Population

A cross-sectional hospital-based study was conducted among patients with bronchial asthma in the pulmonology clinic of Muhimbili National Hospital in Dar-es-salaam, Tanzania from September 2018 to January 2019. This site was chosen purposefully owing to its relative large number of asthma patients compared to other hospitals in Dar es Salaam. Pulmonology clinic runs once a week for public patients and daily for private patients. On average, about 20 asthma patients are attended to in the public clinic per week and 5 to 10 in the private clinic daily. In this clinic, nearly all asthma patients were on at least one Pressurised Metered Dose Inhaler (pMDI). A few patients were using powder inhalers interchangeably with pMDI depending on availability and income level. Additionally, some patients were on various oral medications for asthma such as; prednisolone, montelukast, aminophylline, antihistamines and medications for other comorbidities. Participants were eligible if they were aged 18 years or older, were outpatient, able to give informed consent and had a physician's diagnosis report positive for asthma ≥ 6 months prior to this study and had been using pMDI for asthma medications for at least 6 months.

### Sample Size Estimation

Sample size was calculated using Kish and Leslie formula (1965). A minimum sample size of 385 was calculated to achieve 80% power for detecting medication adherence of at least 49.4%^23^ at a significance criterion of *α* = 0.05.

### Study Procedures

Participants were identified consecutively and, if they met the eligibility criteria and provided consent, were enrolled until the required sample size was reached. The study screen 418 participants to obtain 385 eligible participants. Face-to-face interview-based on a structured questionnaire was conducted by trained physicians to extract patient's data including; age, gender, educational level, occupation, co-morbidity, smoking habit, health insurance coverage, medication used for asthma therapy, alternative medication for asthma apart from inhalers, duration of using inhaled medication, duration of asthma and if previously received training on pMDI use from any health personnel. Other factors that were enquired included; difficulty with medication refills, forgetfulness, fear of side effects and difficulty in inhaler use.

### Assessment of Asthma Control

Asthma control was assessed using a validated Asthma Control Test (ACT),^24^ a 5-questions interview-based questionnaire. The ACT is patient-centred and recalls the patient's experience of 5 items: The effect of asthma on daily functioning, Daytime and nocturnal asthma symptoms, the use of rescue medications, and patient's perception of asthma control over the previous 4 weeks. Each item includes 5 response options corresponding to a 5-point rating scale. Responses for each of the 5 items were summed to yield a score ranging from a minimum of 5 (poor control of asthma) to a maximum of 25 (complete control of asthma). The scores are categorised into 3. A score of 25 corresponds to totally controlled, 20 to 24 (well controlled) and <20 (uncontrolled). In the present study, participants who scored ≥20 were classified as well controlled while those who scored 19 and less were regarded as having poorly controlled asthma.

### Assessment of Inhaler Medication Adherence

Inhaler adherence was assessed using a validated 10-item Test of Adherence to Inhalers (TAI) questionnaire.^25^ The TAI questionnaire includes a patient domain that is self-administered and scored from 1 to 5 (1 = worst possible score; 5 = best possible score). The total score for the questionnaire ranges from 10 to 50. Adherence is rated as good (score: 50), intermediate (score: 46–49), or poor (score: <45).^25^ This questionnaire was administered as an interview.

### Assessment of Inhaler Technique

All participants were required to demonstrate how they normally use their inhaler. To attain this, placebo pMDI was provided to each participant who was then asked to demonstrate how to use it. All demonstrated steps of the placebo pMDI use was scored against a standardised inhaler checklist.^26^ The checklist composed of 10 steps derived from previously published inhaler checklist and manufacture's recommendation. Each correctly performed step was given a value of one, whereas, non-performed or incorrectly performed step was given a value of zero. The participant's technique was judged to be good if all the steps on the checklist for correct inhaler use were performed accurately and poor if any of the recommended step(s) were/was missed out or performed inaccurately.

### Ethical Approval and Consent to Participate

The Institutional Review Board of the Muhimbili University of Health and Allied Sciences gave ethical clearance for the study. The conduction of the study was in accordance to Helsinki's declaration. All participants gave an informed consent before enrolment. No participants' identifying data was collected for this study.

### Data Analysis

SPSS version 23 statistical software was used for analysis of the data. Summary statistics were reported as means with standard deviations or median with interquartile range for continuous data as deemed appropriate, and frequencies with percentages for categorical data. Association between predictive variables (socio-demographic and clinical characteristics of the patients) and dependent variable (inhaler medication use classified as adherence or non-adherence) was checked using binary logistic regression. Variables with p-value ≤.20 in univariate model were included in the multivariate model. Adjustments took into account education level, occupation, possession of health insurance, inhaler affordability, fear of side effects, being too busy, forgetfulness and inhaler use technique. Significance level was set at p-value less than .05 for the multivariate model.

## RESULTS

A total of 385 patients were included in the study, 206 (53.5%) were female, 213 (55.3%) were married and 204 (53.0%) had attained secondary level education. Nearly half of the participants (49.1%) were self-employed. The majority (93.5%) were non-smokers. More than half of the respondents (53.8%) had healthy insurance coverage while 33.2% had at least 1 co-morbidity condition. Most of the participants (79.5%) had been diagnosed with asthma for more than 5 years and had used inhalers for more than 2 years (55.6%) (Table 1). The median (IQR) adherence score was 44.0 (42-48). The minimum score was 19 and maximum score 50. Only 153 of the 385 respondents (39.7%) were adherent to inhalers.

**TABLE 1:** Socio-Demographic and Clinical Characteristics of Asthmatic Patients Attending MNH Pulmonology Clinic (N=385)

Character	Frequency (n)	Percent (%)
**Age (years)**		
18-29	138	35.8
30-39	80	20.8
40-49	72	18.7
50-59	59	15.3
≥60	36	9.4
**Sex**		
Male	179	46.5
Female	206	53.5
**Marital status**		
Single	131	34.0
Married	213	55.3
Divorced/separated	19	5.0
Widowed	22	5.7
**Education level**		
No formal education	6	1.5
Primary school education	143	37.6
Secondary school education	204	53.0
College/University education	32	8.3
**Occupation**		
Unemployed	84	21.8
Self employed	189	49.1
Employed	66	17.1
Student	46	11.9
**Smoking habit**		
Former smoker	24	6.2
Current smoker	1	3.0
Never smoker	360	93.5
**Possession of a health insurance**		
Yes	207	53.8
No	178	46.2
**Having co morbidity**		
Yes	128	33.2
No	257	66.8
**Duration of asthma**		
≤5 years	79	20.5
>5 years	306	79.5
**Duration of inhaler use**		
≤2 years	171	44.4
>2 years	214	55.6

**FIGURE 1:**
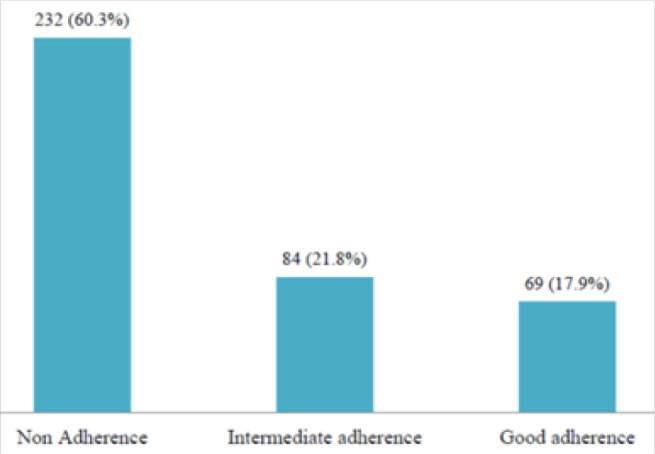
Inhaler Adherence Levels among Asthma Patients Attending MNH Pulmonology Clinic

Figure 1 shows the level of inhaler adherence among the participants. Good inhaler adherence was observed in 69 (17.9%) participants while 84 (21.8%) had intermediate adherence and 232 (60.3%) were non-adherent to inhalers.

Table 2 shows the Logistic regression of factors affecting adherence to inhalers among the study participants. After controlling for other factors, the odds of inhaler non-adherence were 4 times higher among participants with no health insurance than those with health insurance, aOR (95%CI)= 4 (2.1-7.8), p<.001. Respondents who had fear of side effects for inhalational medications had 8.2 times increased odds of non-adherence compared to those who had no fear, aOR (95%CI)=8.2 (4.2-16), P <.01. The odds of inhaler non-adherence were about 6 times more among respondents who were too busy than those who weren't, aOR (95%CI) = 5.9 (1.5-22.4), P<.01. Participants who had alternative medications for asthma apart from inhalational medication had 2.1 times increased odds of non-adherence compared to those who had no alternative medications, aOR (95%CI)=2.1 (1.6-6.2), P <.01. Patients who had incorrect inhaler technique had 12 times increased odds of non-adherence compared to those with good technique aOR (95%CI)=12.1 (5.1-26.5), P<.001. Unavailability of inhalers nearby once one has been finished; unaffordability, forgetfulness and report of difficulty in use of inhalers were not associated with inhaler non-adherence.

**TABLE 2:** Logistic Regression of Factors Affecting Adherence to Inhalers among Asthma Patients Attending MNH Pulmonology Clinic (N=385)

Factor	cOR	p-value	aOR	p-value
**Age in years**				
≤40	1		1	
>40	0.7 (0.5-1.1)	0.14	0.6 (0.3-1.2)	0.14
Sex				
Female	1			
Male	1.0 (0.6-1.4)	0.81		
**Marital status**				
Married	1		1	
Single/divorced/separated	1.5 (0.9-2.5)	0.08	1.6 (0.8-2.9)	0.16
**Education level**				
Secondary or higher	1	1		
Primary or lower	1.6 (1.1-2.5)	0.02	0.6 (0.2-1.8)	0.39
**Duration of asthma**				
≤5 years	1		1	
>5 years	1.5 (0.9-2.5)	0.09	2.1 (0.9-4.6)	0.07
**Comorbid conditions**				
Yes	1			
No	0.8 (0.5-1.3)	0.39		
**Possession of health insurance**				
Yes	1	1		
No	4.1 (2.6 – 6.4)	<0.001	4.0 (2.1 – 7.8)	<0.001
**Inhaler finished and was not available nearby**				
No	1			
Yes	2.2 (1.05 – 4.9)	0.037	2.6 (0.9 – 7.7)	0.072
**Can't afford to buy one**				
No	1			
Yes	6.3 (1.4 – 27.7)	0.014	3.2 (0.48 – 1.4)	0.226
**Fear of side effects**				
No	1			
Yes	4.07 (2.5 – 6.4)	<0.01	8.2 (4.2 – 16)	<0.01
**Use of alternative medication**				
No	1		1	
Yes	2 (1.8 – 5.1)	<0.01	2.1 (1.6 – 6.2)	<0.01
**Inhaler technique**				
Correct use	1		1	
Incorrect use	15.6 (9.1-26.5)	<0.001	12.1 (5.1-26.5)	<0.001

cOR=Crude odds ratio, aOR = adjusted odds ratio.

**FIGURE 2:**
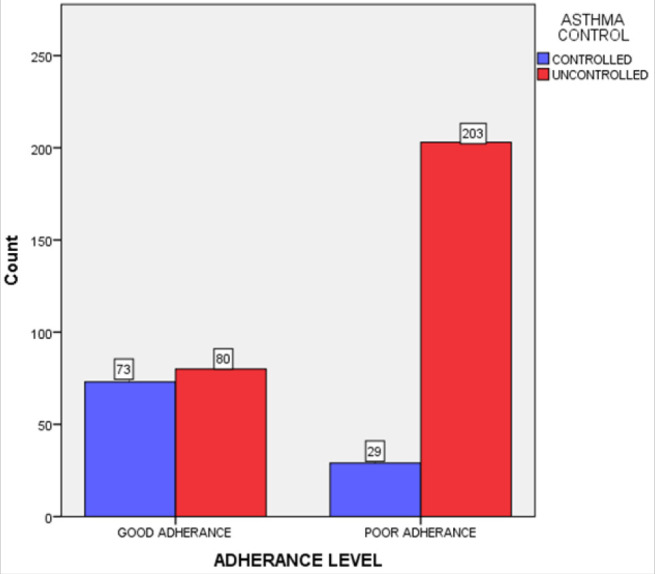
Relationship between Inhaler Non-Adherence and Asthma Control among Asthma Patients Attending MNH Pulmonology Clinic

Figure 2 shows the level of asthma control in relation to inhaler non-adherence. Using the ACT questionnaire, only 102 of the 385 participants (26.5%) had good asthma control. Significantly more patients 73(47.7%) had good asthma control among those with good or intermediate adherence to their inhalers (N=153) than among those with poor adherence 29 (12.5%), (N=232), p<.001.

Majority of patients with secondary level education (65.8%) or college/university level (100%) had insurance cover compared to those with primary level (36%) or no formal education (50%), P<.001. (Table 3)

**TABLE 3:** Possession of insurance Cover by Level of Education among Asthma Patients in Muhimbili National Hospital, N=385

Education level	Total number	Possession of an insurance cover Yes Number (%)	P value^*^
No formal education	6	3 (50)	
Primary level education	189	68 (36)	
Secondary level education	158	104 (65.8)	
College/uniersity level	32	32 (100)	< 0.001

* P value by Fisher's Exact test

## DISCUSSION

This study investigated inhaler adherence, asthma control and factors affecting non-adherence. The relationship between inhaler adherence and asthma control was also investigated. The study observed a high inhaler non-adherence rate of 60.3% and a low asthma control rate of 26.5%. Factors associated with poor inhaler-adherence in the studied population were; primary or lower level education, unemployment, lack of health insurance, fear of medication side effects, being too busy or forgetful, having an alternative medication for asthma and having incorrect inhaler use technique. Most of these factors that affect inhaler adherence and thus asthma control can be modified or addressed to improve inhaler adherence and asthma control. The rate of inhaler non-adherence in the present study is comparable to previously reported adherence rates from Ethiopia (50.6%),^23^ Egypt (71.7%)^27^ and India (61.5%).^28^ Lower rates have been reported in a systematic review conducted by Barnes et al, non-adherence to inhalers was found to range from 22% to 63%.^10^

With regards to factors affecting inhaler adherence in the present study, lack of health insurance negatively impacted inhaler adherence. This might inform on medication affordability in which case, lack of health insurance translates to inability to buy medication out-of-the pocket. Results of previous studies showed that limited coverage for medications and out-of-pocket costs affect prescription initiation and consistent medication use.^23,29,30^ This cost-related non-adherence had an impact to patients from minority populations with limited incomes.^23,29,30^ A study conducted in Pakistan reported that 30.7% of patients were non adherent merely due to medication costs.^31^

Inhaler non-adherence among participants with low education achievements have also been reported in other similar studies.^27,31^ Patients with low education achievement are more likely not to have health insurance thus predisposed to medication cost-related non-adherence, particularly in countries with no universal health insurance coverage. Furthermore, participants with low education might not be as knowledgeable about their health conditions as those with high education.

Fear of medication side effects is reported in other studies, However, this is explained to be unjustifiable since the fear does not originate from actual experience of the side effect.^23,32^ A comparison study to evaluate patients' reported side effects with clinician's estimates found that patients were over reporting the side effects compared to actual prevalence reported by clinicians.^33^ A meta-analysis to assess adherence-related beliefs showed that adherence to medication was influenced by the patients’ beliefs and judgment about their personal need for the prescribed medication relative to their concerns about the potential adverse effects.^34^ Fear of inhaler medication as well as preference to use alternative medications for asthma can also originate from lack of knowledge about inhalers compared to other treatment options.^35^

Studies have reported that the preferred alternative drugs were oral aminophylline, salbutamol and herbal medications. Other studies have also shown an association between the use of alternative medications for asthma management and non-adherence.^7,33^ This preference to alternative medication could be explained by lack of knowledge on asthma and its management as well as belief on oral medication that they are safer than inhalational medications. Most participants in this study reported that the reasons for use of alternative medications were; lack of symptoms relief with inhaler use (although this information was not systematically collected), cost of inhaler and unavailability of inhaler medication in some parties of the country.

Participants who reported to be too busy with their daily activities were non adherent to inhaler medication as compared to those who weren't too busy. In this study, nearly a half (49.1%) of the participants reported to be self-employed. With their busy schedule on daily basis in order to generate income, they turned non-adherent. Previous studies have shown that busy lives coupled with negative perceptions of medication and inhaler taking were the main reasons for forgetfulness and hence non adherence.^31,36,37^

Additionally, incorrect inhaler technique was associated with non-adherence. In this study, among the non-adherent group, majority (71.6%) demonstrated incorrect inhaler technique. Incorrect inhaler technique leads to poor drug delivery to the lungs and hence poor asthma symptoms control which may predispose a patient to non-adherence.^38^ Incorrect inhaler technique seen in these patients might originate from lack of regular training on inhaler use and infrequent monitoring of its use by their physicians.

### Study Limitations

The checklist used for inhaler technique assessment varies among researchers as well as inhaler manufacturer recommendations. Although the steps are more or less the same, they are not standardised. This could have magnified the error rates among study participants.

The consecutive enrolment of the participants might have introduced selection bias inclining more onto selecting those with poor inhaler adherence, thus poor asthma control.

Due to its cross-sectional design, no conclusions on causality can be drawn if non-adherence was the cause of poor asthma control or the vice versa.

## CONCLUSION

Non-adherence to inhaler medication and poor asthma control were common among participants. Lack of insurance coverage significantly affected adherence to inhalers. Concurrent use of oral medications for asthma negatively affected inhaler adherence. The undue fear of inhaler medication side effects were significant. Emphasis on patients' education in asthma clinics will improve adherence level, since this will eliminate myths and undue fears about inhalers and bring about eventual attainment of optimum asthma control.
